# A Case of Placental Mesenchymal Dysplasia

**DOI:** 10.1155/2013/265159

**Published:** 2013-11-20

**Authors:** Shigeki Taga, Junko Haraga, Mari Sawada, Aya Nagai, Dan Yamamoto, Ryoji Hayase

**Affiliations:** Department of Obstetrics and Gynecology, National Hospital Organization Fukuyama Medical Center, 720-0825 Okinogamicho, Fukuyama City, Hiroshima Prefecture 4-14-17, Japan

## Abstract

Placental mesenchymal dysplasia (PMD) rarely complicates with pregnancy. A 30-year-old woman, gravida 3, para 3, presenting with placentomegaly, was referred to our department at 18 weeks of gestation. An ultrasonography revealed a normal fetus with a large multicystic placenta, measuring 125 × 42 × 80 mm. The border between the lesion and normal region was not clear. Color doppler revealed little blood flow in the lesion. Magnetic resonance imaging revealed normal fetus and a large multicystic placenta. Serum human chorionic gonadotropin level was 20124.97 U/L, which was normal at 20 weeks of gestation. Thus, placental mesenchymal dysplasia rather than hydatidiform mole with coexistent fetus was suspected. Then, routine checkup was continued. Because she had the history of Cesarean section, an elective Cesarean section was performed at 37 weeks of gestation, and 2520 g female infant with apgar score 8/9 was delivered. The baby was normal with no evidence of Beckwith-Wiedemann syndrome. Placenta of 20 × 16 × 2 cm, weighing 720 g, was bulky with grape like vesicles involving whole placenta. Microscopic examination revealed dilated villi and vessels with thick wall which was lacking trophoblast proliferation. Large hydropic stem villi with myxomatous struma and cistern formation were seen. PMD was histopathologically confirmed.

## 1. Introduction

Placental mesenchymal dysplasia (PMD) is a rare benign entity characterized by placentomegaly and grape like vesicles resembling molar pregnancy on ultrasonography. We diagnosed a case of PMD at the second trimester and obtained healthy baby. Herein, we report the case and review the literature.

## 2. Case Presentration

**Figure 1:**
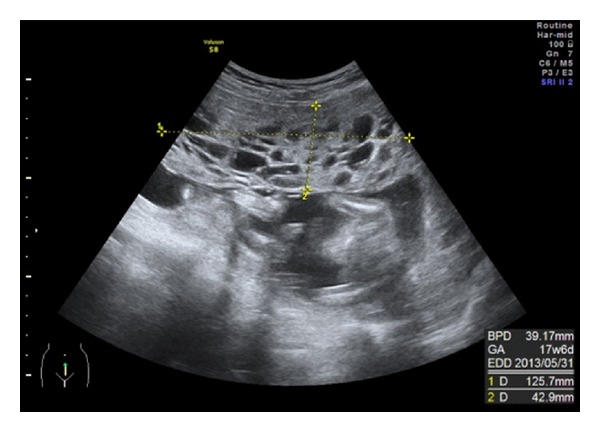
An ultrasonography showing a large multicystic placenta.

A 30-year old woman, gravida 3, para 3, with a previous Caserean delivery presenting with placentomegaly was referred to our department at 18 weeks of gestation. Her medical history was significant for myomectomy at the age of 18. An ultrasonography revealed a normal appearing fetus and a large multicystic placenta measuring 125 × 42 × 80 mm. The border between the lesion and normal region was not clear (Figure 1). 

**Figure 2:**
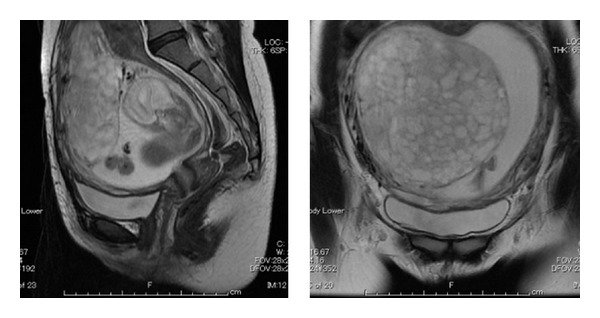
Magnetic resonance imaging showing a large multicystic placenta at 20th weeks of gestation. Normal part of the placenta was not clearly identified.

A chorioangioma was suspected first, but color Doppler imaging revealed little blood flow. Then the magnetic resonance imaging was performed at 20 weeks of gestation. A normal fetus and a large multicystic placenta were seen but normal part of the placenta was not clearly identified (Figure 2).

**Figure 3:**
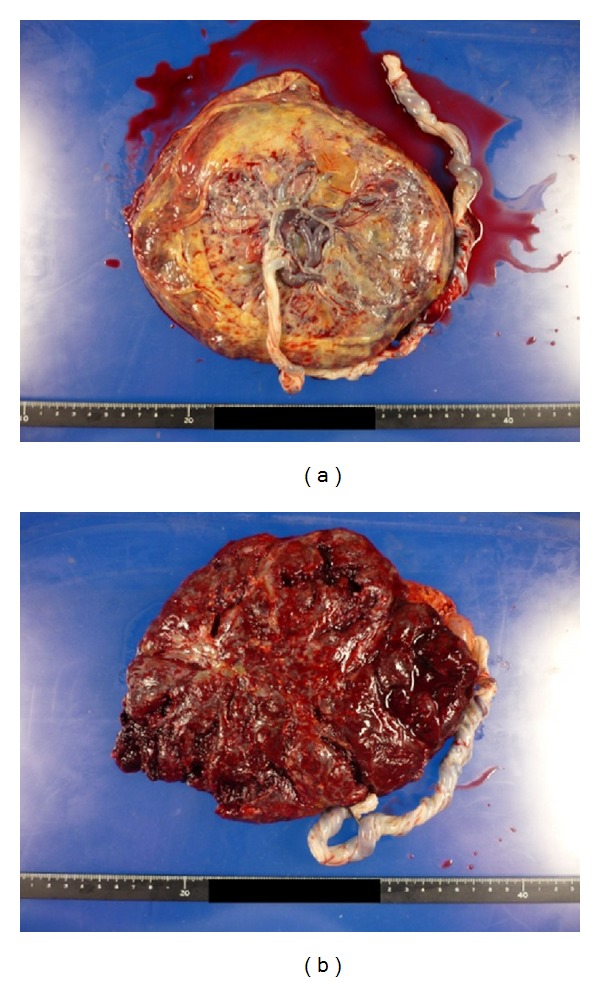
Placenta of 20 × 16 × 2 cm, weighing 720 g, was bulky with grape like vesicles involving whole placenta. (a) Fetal plate and (b) maternal surface.

Omphalocele was not identified. Serum human chorionic gonadotropin level was 20124.97 U/L, which is normal at 20th weeks of gestation, and we concluded that PMD was more probable rather than hydatidiform mole with coexistent fetus. The karyotype was not examined. Then routine checkup was continued. Because she had the history of Cesarean section, Cesarean section was performed at 37 weeks of gestation. Under spinal anesthesia, a 2520 g female infant with Apgar score of 8 and 9 at 1 and 5 min, respectively, was delivered. The infant had no evidence of Beckwith-Wiedemann syndrome. Placenta of 20 × 16 × 2 cm, weighing 720 g was bulky with grape like vesicles involving whole placenta with no border (Figure 3).

**Figure 4:**
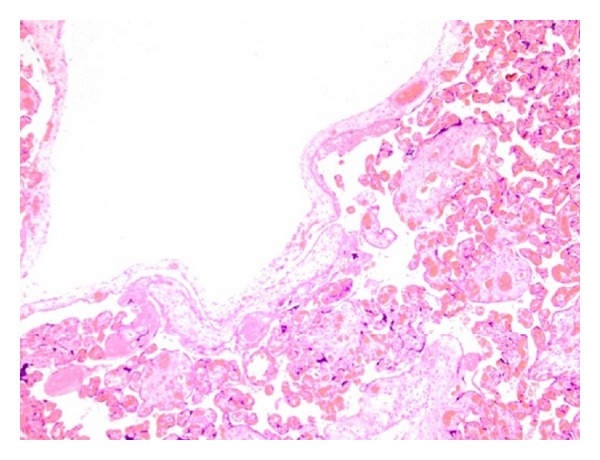
Enlarged villi and vessels with thick wall which is lacking trophoblast proliferation and large hydropic stem villi with myxomatous struma forming cistern were observed (H.E. × 100).

The cord was 56 cm in length and 1 cm in diameter and appeared normal. Microscopic examination revealed enlarged villi and vessels with thick wall which was lacking trophoblast proliferation. Large hydropic stem villi with myxomatous struma forming cistern were observed (Figure 4). 

The diagnosis of PMD was confirmed. Her postoperative course was uneventful. 

## 3. Discussion

PMD is a rare benign entity characterized by placentomegaly and grape like vesicles resembling molar pregnancy on ultrasonography [1]. PMD was initially described by Moscoso et al. in 1991 as placentomegaly which gives the image of partial hydatidiform mole with elevated level of alpha feto-protein [2]. Approximately 100 cases have been reported since then. The cause of this clinical entity is currently still unknown. All the reports are episodic and no systemic research has been done. Nayeri et al. reviewed 61 cases and reported that 50% revealed placentomegaly and 80% cystic placenta [3]. True incidence is not known but it has been estimated at 0.02% [4]. However, Zeng et al. [5] identified only two cases in 95000 deliveries at their institution. They reported the incidence of 0.02 per 1000, which was lower than that reported before by Arizawa and Nakayama [4].

Approximately 23% of the cases of PMD are associated with Beckwith-Wiedermann syndrome, which is characterized by macrosomia, exomphalos, macroglossia, omphalocele, internal visceromegaly, placentomegaly, and increased childhood tumors. Qichang et al. reported a case of PMD concomitant with chorioangioma [6]. Nayeri et al. stated in their review of 61 cases that pregnancy complication included intrauterine growth restriction (IUGR; 33%), intrauterine fetal demise (IUFD; 13%), and preterm labor (33%) [3]. Differential diagnosis includes complete molar pregnancy with cotwin, a partial molar pregnancy, and chorioamgioma. Human chorionic gonadotrophin is useful for differential diagnosis. Although abnormal high level of hCG usually indicates molar disease, some molar pregnancies reveal normal level. 

Placentomegaly is usually presented and chorionic vessels were aneurysmatic and dilated. Many pathologists are unfamiliar with PMD, which may lead to underdiagnosis of this entity. Differential diagnosis from gestational trophoblastic disease is important because management and outcomes are different. Histopathologically, trophoblast proliferation is revealed in molar pregnancy but not in PMD. Partial molar pregnancy is accompanied by an abnormal triploid fetus and was not strongly suspected in this case because fetal development was normal. In case of partial molar pregnancy, pregnancy termination is indicated. A triploid fetus usually has several morphological anomalies and tends to die in the first trimester. When there is the combination of a molar appearance to the placenta and a sonographically normal fetus, suspicion for PMD may be particularly increased. In PMD, blood test reveals elevated AFP while *β*-hCG slightly increases. On ultrasonography, the placenta of complete molar pregnancy with cotwin and partial molar pregnancy look heterogeneous, with partially solid and cystic areas [7, 8]. Cohen et al. [9] reported that the karyotype was normal in 32/36 (89%) and stressed that the diagnosis of PMD should not be disregarded when an abnormal fetus and/or an abnormal karyotype are demonstrated. Ohyama et al. [10] reported that noodle like structure indicates dilated large hydropic stem villi and is characteristic of PMD. 

PMD is rare and resembles molar pregnancy, but the prognosis is different. The fetuses are normal in the majority of cases. This clinical entity should be kept in mind to avoid unnecessary termination of pregnancy.
